# Outcomes and the effect of PGT-M in women with hormone-related hereditary tumor syndrome

**DOI:** 10.3389/fonc.2024.1378019

**Published:** 2024-05-10

**Authors:** Dingran Wang, Xueling Song, Xiaohui Zhu, Liying Yan, Xu Zhi, Jie Yan, Huamao Liang, Jie Qiao

**Affiliations:** ^1^ Center for Reproductive Medicine, Department of Obstetrics and Gynecology, Peking University Third Hospital, Beijing, China; ^2^ Beijing Key Laboratory of Reproductive Endocrinology and Assisted Reproductive Technology, Peking University Third Hospital, Beijing, China; ^3^ Department of Obstetrics and Gynecology, Peking University Third Hospital, Beijing, China; ^4^ Beijing Advanced Innovation Center for Genomics, Beijing Key Laboratory of Reproductive Endocrinology and Assisted Reproductive Technology, Peking University Third Hospital, Beijing, China

**Keywords:** PGT, hereditary tumor syndrome, fertility preservation, HBOC (hereditary breast and ovarian cancer), lynch syndrome (hereditary non-polyposis colorectal cancer)

## Abstract

**Purpose:**

To review the outcome of PGT-M in hormone-related hereditary tumor syndrome and evaluate the effect of ovarian induction on tumor growth in those patients.

**Methods:**

Medical records of PGT-M were retrospectively analyzed in patients with hormone-related heritage tumors in our reproductive center. A total of eleven women with hereditary breast and ovarian cancer (HBOC) (including BRCA1/2 mutation carriers), and Lynch syndrome (including MMR gene mutation carriers) were included. Thirteen IVF/PGT-M cycles were performed. Eleven for PGT-M and two for fertility preservation. The ovulation protocol, numbers of oocytes retrieved and two pronuclei (2PN) zygotes, PGT-M results, and clinical outcomes were analyzed. Tumor progression was also estimated by comparing transvaginal ultrasound (TVS), MR, CT, or colonoscopy according to the follow-up requirements of different tumors.

**Results:**

Eleven IVF/PGT-M cycles were performed with an antagonist protocol; Two cycles were performed with a mild stimulation protocol. The total dose of gonadotropin (Gn) was 1827 IU per patient (range from 1200 to 2625 IU). The median number of oocytes retrieved was 13 (range from 4 to 30), and the median number of 2PN zygotes was 8 (range from 2 to 16). A total of 32 embryos underwent PGT-M, and 9 (28.1%) embryos were suitable for transfer. Six transfer cycles were performed, and 5 cycles got clinical pregnancy (83%) with five newborns (83%). The follow-up examinations conducted 10-18 months after PGT-M/delivery revealed no new lesions or tumor progression.

**Conclusion:**

PGT-M results can provide important information for improving the consultation of hormone-related heritage tumor patients regarding their fertility preservation and reproductive options. Ovarian induction for women with hormone-related hereditary tumor syndrome is not associated with tumor progression.

## Introduction

Heritage tumor syndrome accounts for 5-10% of all cancer cases. Although each syndrome shows very specific clinical symptoms, heritage tumor syndromes have some common features, such as involving multiple and paired organs like bilateral breast cancer and bilateral renal cell carcinoma. The onset age is often 10-15 years earlier than usual, and close relatives may have the same or associated tumors (1). Most hereditary tumor syndrome is autosomal dominant inheritance, involving genetic variants inheritance and germline mutations of oncogenes and tumor suppressor genes with a 50% risk transmission to offspring.

To date, more than 60 types of hereditary tumor syndromes have been identified, involving approximately 70 genes (2). The main hereditary tumor syndromes that involve gynecologic cancers include hereditary breast and ovarian cancer (HBOC) syndrome and Lynch syndrome. HBOC is caused by mutations of tumor suppressor genes *BRCA1* (17q21.31) or *BRCA2*(13q13.1) and is associated with higher risk of breast and ovarian cancer (3). BRCA mutation carriers have a cumulative lifetime incidence up to 72% for breast cancer and 44% for ovarian cancer (4). Beyond BRCA mutated HBOC, several homologous recombination-related genes such as BRIP1, RAD51C/D, PALB2, and ATM may also increase ovarian cancer risk with moderate-penetrance (5, 6). Lynch syndrome, also named as hereditary non-polyposis colon cancer(HNPCC), is caused by the germline mutations of four mismatch repair (MMR) related genes, including *PMS2, MSH6, MLH1*, and *MSH2*. Lynch syndrome increases the risk of endometrial and ovarian cancer, as well as colorectal, urothelial tract, stomach, and small bowel carcinoma (7). It has been reported that the cumulative risk of inherited digestive cancers by the age of 70 was 46% for *MLH1*, 48% for *MSH2* and 23% for *MSH6* mutation (8), while the cumulative risk of endometrial cancer was 34% for *MLH1*、51% for *MSH2*、49% for *MSH6* and 24% for *PMS2* (9). Other relatively rare syndromes associated with gynecologic cancers include Lefameni syndrome, Cowden syndrome, Peutz-Jeghers syndrome, DICER1 syndrome and rhabdoid tumor predisposition syndrome 2 (10). These hereditary tumors involved with gynecologic cancers are suspected related with hormone levels, including estrogen and progesterone (3).

Patients with hereditary tumor predisposition syndromes involved gynecologic cancer usually need to receive adjuvant therapies like chemotherapy and/or radiotherapy, which both adversely impact reproduction. It is challenging to predict the fertilization capacity of these patients. Freezing of oocytes, ovarian tissues, and embryos may preserve fertility among cancer patients. If patients wish to prevent the genes from being passed to their offspring, they should first receive the evaluation by Multidisciplinary Team (MDT) consists of oncologists, geneticists and reproductive medicine experts. After considering the pathology and penetrance of mutated genomic variants, family history and other cancer-related risk factors, they can choose prenatal diagnosis during pregnancy or preimplantation genetic testing for monogenic/single gene defects (PGT-M) to achieve genetic blockade.

Limited data is available about how the results of PGT-M influence the progression and recurrence, as well as reproductive outcomes of patients with hormone-related heritage tumor syndromes. Several studies have analyzed the impact of *in vitro* fertilization (IVF) on HBOC patients or BRCA1/2 mutation carriers (11), but PGT-M cycles and fertility preservation were not investigated in these studies. In PGT-M cycles/fertility preservation cycles, increased doses of gonadotropins (Gn) are used to obtain more oocytes, increasing the concentration of estradiol, which may theoretically increase the risk of recurrence of cancer syndromes (12). In this study, we retrospectively analyzed the impact of PGT-M results on the clinical outcomes of patients with hormone-related heritage tumors in our reproductive center and estimated tumor progression by comparing MR, CT, or colonoscopy according to different tumor follow-up requirements after IVF or pregnancy.

## Materials and methods

### Study design and study definitions

We retrospectively reviewed patients who received PGT-M treatment between 2015 and 2023 in the tertiary university-affiliated medical center Peking University Third Hospital(PUTH). Patients were diagnosed with hormone-related heritage tumor syndromes including HBOC, Lynch syndrome. They had received examination by the corresponding specialist. These patients received PGT-M or fertility preservation treatment at the reproductive center of PUTH.

Patients inclined to receive PGT-M or fertility preservation were evaluated by the MDT team of obstetricians, geneticists, reproductive experts, and oncologists. Five Lynch syndrome patients, and six HBOC patients(including carriers) were finally included in the study. All patients signed informed consent forms, and the hospital ethics committee with No.2008013 approved the study.

We collected the patients’ general information, obstetric history, and hereditary tumor history. Their surgery, chemotherapy information, and major comorbidities were recorded. PGT-M information was analyzed, including ovarian stimulation protocol, total dose of gonadotrophins, endometrium thickness, days of stimulation, use of letrozole and tamoxifen, as well as number of oocytes retrieved, number of oocytes fertilized, and number of embryos underwent PGT-M diagnosis, and number of clinical pregnancy and live birth. Ultrasonography indicating intrauterine gestational sac or fetal pole was defined as a clinical pregnancy. The clinical pregnancy rate or live birth rate was calculated per transfer.

### Establishment of PGT-M analysis systems

Prior to PGT-M, the familial gene mutations were validated in genomic DNA samples isolated from the peripheral blood of carrier patients. The pedigrees’ DNA and the embryo’s biopsied sample were amplified with Multiple Annealing and Looping Based Amplification Cycles (MALBAC) and sequenced at 2X coverage. The sequenced reads were mapped to the human reference genome using Burrows-Wheeler Aligner (BWA) software with default settings, and the copy number variation of each embryo was identified with Hidden Markov Model (HMM) copy software. To determine the mutation carrier status of each embryo, whole chromosome haplotyping analysis was performed with scHaplotyper software (13). Finally, the target sites were validated by Sanger sequencing.

### Oncological follow-up after PGT-M/fertility preservation

The oncological outcomes of all the patients who received PGT-M or fertility preservation procedures were investigated by physical examination and imaging examination according to the tumor follow-up project. For Lynch syndrome patients, colonoscopy and transvaginal ultrasound (TVS) were performed for follow-up. For HBOC patients, TVS, breast ultrasound, CT or MRI was conducted for evaluation.

### Statistical analysis

SPSS 25 were used to perform statistical analysis of the data (SPSS 25, Chicago, IL, USA). Normally distributed variables are described with standard deviations, and nonnormally distributed variables are expressed as medians and 25th and 75th percentiles. Nonparametric test was used for nonnormal distribution data. P<0.05 indicates a statistically significant difference.

## Results

### Patients characteristics

A total of eleven women with hormone-related hereditary tumors were included, consisting of five Lynch syndrome, and six HBOC patients including carriers. The average maternal age at the time of IVF/PGT-M was 32.18 ± 2.93y (range from 27-37y), two cycles were performed in women with advanced maternal age (≥35 years). The average AMH was 4.37 ± 2.42 ng/ml (1.56-8.68ng/ml). All of these patients did not have an infertility history. General information and tumor history of patients are displayed in **Table 1**.

**Table 1:** General information and tumor history of patients with familial neoplasms.

Patient number and fertility treatment	Age	Gene	Disease	Surgery	Chemotherapy	Symptoms & comorbidities	AMH(ng/ml)
**PGT-M1**	37	MLH1 c.793C>T	Lynch carrier, multiple colonic polyps	Colonoscopy polypectomy	None	None	2.71
**PGT-M2**	32	MSH6 c.2095G>T	Lynch carrier	None	None	None	4.65
**PGT-M3**	35	MLH1 c.184C>T	Lynch, colorectal carcinoma	Colectomy	√	Bowel obstruction	2.67
**PGT-M4**	33	MLH1 c.755C>A MSH6 c.4065_4066insTTGA	Lynch colorectal carcinoma	Colectomy	None	None	6.73
**PGT-M5**	33	MSH2 c.2211-2A>C	lynch carrier, multiple colonic polyps	Colonoscopy polypectomy	None	None	1.84
**PGT-M6**	33	BRCA1 c.3916-3917del	Carrier, breat nodules (BI-RADS3)	None	None	None	8.68
**PGT-M7**	32	BRCA2 c.9439del	Breast cancer	breast-conserving surgery	None(endocrine therapy)	None	2.41
**PGT-M8**	28	BRCA1 c.2572C>T	Carrier	None	None	None	6.86
**PGT-M9**	34	BRCA2 c.4415_4418del	Carrier, breast nodules (BI-RADS3)	None	None	None	1.56
**FP1**	27	BRCA2 c.4318delA	Breast cancer	breast-conserving surgery	None	None	6.36
**FP2**	30	BRCA1 c.1961dup	Breast cancer	breast-conserving surgery	None	None	3.62

Thirteen IVF/PGT-M cycles were performed, including ten cycles for PGT-M, one BRCA2 carrier patient gave up the PGT-M after oocytes retrieval in her second cycle and underwent a frozen embryo transfer. Another two patients received fertility preservation. Patients had a median cycle number of one cycle. Eight patients received one cycle, two had two cycles.

Five patients (PGT-M1~PGT-M5) were diagnosed with Lynch syndrome or MMR gene mutation carrier. They all had familiar traits. PGT-M1 was found to be a carrier of the MMR genetic variant. The patient’s father, the family proband, was diagnosed with colon cancer at the age of 50 and underwent surgical treatment. Genetic tests conducted on both tumor tissue and blood samples from the father revealed a pathogenic site MLH1: c.793C > T mutation, which was also detected in the patient. The patient was found to have multiple polyps in the colon by colonoscopy, but TVS revealed no abnormality.PGT-M2 found herself a carrier of MMR genetic variant after her father was diagnosed with colon cancer at the age of 46. The patient had a likely pathogenic site MSH6:c.2095G>T mutation, the same as her father. She took the evaluation half a year before IVF and found no abnormality by colonoscopy and TVS. Patient PGT-M3 underwent laparoscopic colon tumor resection and chemotherapy in 2017, with her genetic testing suggesting MLH1: c.184C>T pathogenic mutation. Her mother underwent ileal surgery without genetic testing. Patient PGT-M4 also had laparoscopic radical resection of colon cancer without chemotherapy in 2017 and was tested for MLH1:c.755C>A and MSH6:c.4065_4066insTTGA mutations, both pathogenic genetic variant. Her father and grandmother both had colon cancer. Patient PGT-M5 had a typical family history of Lynch syndrome. Her mother had colon cancer at the age of 54 and endometrial cancer at 56. Her grandfather had colon cancer at 50, while his sister had endometrial cancer at 58. Patient PGT-M5 had a MSH2:c.2211-2A>C pathogenic mutation, the same as her mother. The patient had no morbidity now, her colonoscopy showed multiple colonic polyps in 2021.

Six patients with BRCA1/2 mutation were included. Patient PGT-M6 had a c.3916-3917del pathogenic mutation, the same as her mother and her aunt, who both had ovarian cancer at 46 and 52. The patient’s breast ultrasound showed multiple BI-RADs3 nodules with the largest size of 1.25*0.41cm before IVF. Patient PGT-M7 had a modified radical resection of the right breast for breast cancer by 2018. She then undertook endocrine therapy (toremifene and Goserelin) for 5 years, her mother and aunt both had breast cancer, her grandmother had esophageal cancer. She had a likely pathogenic BRCA2: c.9439del mutation. PGT-M8 was a BRCA1:c.2572C>T pathogenic mutation carrier, same as her mother who had breast cancer at 45 and passed away at 49. Her grandfather had a colon cancer and her aunt had a cervical cancer. BRCA2 mutation(c4415-4418del, pathogenic) of patient PGT-M9 was identified through a tumor genetic screening. Her father and father’s sister both had the same mutation but had no breast or cancer history. The first PGT-M cycle did not harvest transferable embryos. She gave up the PGT-M after oocytes retrieval in her second cycle and underwent a frozen embryo transfer, resulting in a live birth. The other two young BRCA1/2 mutation patient underwent one ovarian induction cycle to preserve fertilization soon after breast cancer surgery and prior to chemotherapy.

### PGT-M cycles

Eleven IVF/PGT-M cycles were performed with an antagonist protocol; Two cycles were performed with a mild stimulation protocol. The total dose of Gn was 1827 IU per patient (range from 1200 to 2625 IU), with stimulation days of 9 days (range from 6 to 13 days). PGT-M results are displayed in **Table 2**. The median number of oocytes retrieved was 13 (range from 4 to 30), and the median number of 2 pronuclei(PN) zygotes was 8 (range from 2 to 16). The median number of biopsied blastocytes per cycle was 3 (ranging from 1 to 5). Total oocyte number was 172, of which 134 developed into MII oocytes. After performing intracytoplasmic sperm injection, 87 2PN zygotes were matured. PGT-M were performed on 32 blastocytes. 9 (28.1%) embryos had no pathogenic gene and chromosome abnormality and were suitable for transfer. Six transfer cycles were performed, and 5 cycles got clinical pregnancy (83%) with five newborns (83%). Results and outcomes between carriers and morbidities was also compared. Although the numbers of oocytes retrieved, MII, 2PN and biopsied blastocytes of morbidities seemed higher than those of carriers, there were no obvious statistical difference due to the small sample size (**Table 3**).

**Table 2:** PGT-M results and outcomes.

	Cycle	Protocol	Total Gn dose (U)	Days of stimulation	LE/ TMXF	Oocytes	MII oocytes	2PN oocytes	Biopsied blastocytes	embryo suitable for transfer	Outcomes
**PGT-M1**	1	Antagonist	2400	8	×	11	11	9	5	2	Live birth (NC)
**PGT-M2**	1	Antagonist	1350	9	×	19	16	12	4	3	Live birth (NC)
**PGT-M3**	1	Antagonist	1875	9	×	11	8	4	2	0	×
	2	Antagonist	2325	9	×	11	9	8	4	1	Live Birth (NC)
**PGT-M4**	1	Antagonist	1350	10	LE	14	8	3	2	0	×
**PGT-M5**	1	Antagonist	1373	8	×	15	12	11	4	0	×
**PGT-M6**	1	Antagonist	1383	9	×	23	20	16	4	1	Non pregnancy(NC)
**PGT-M7**	1	Antagonist	1800	7	LE	6	5	3	1	0	
**PGT-M8**	1	Antagonist	1200	8	×	30	22	16	4	2	Live birth (NC)
**PGT-M9**	1	Antagonist	2625	13	×	5	3	3	2	0	×
	2	Antagonist	2100	7	×	4	2	2	×	×	Live birth(AC)
**FP1**	1	Mild	1350	6	LE	10	8	×	×		×
**FP2**	1	Mild	2100	7	LE	13	10	×	×		×

NC, natural cycles; AC, artificial cycles; LE, Letrozole; TMXF, tamoxifen; Gn, gonadotropin.

**Table 3:** PGT-M results and outcomes between carriers and morbidities.

	Carrier	Morbidity	P
Cycles	7	6	
Age (y)	33 (32,34)	32.5 (29.3, 35.0)	0.614
AMH (ng/ml)	2.71(1.56, 6.86)	2.67(2.20,6.45)	0.886
Gn dose	1383 (1350,2400)	1837 (1350,2156)	0.943
Oocytes	15 (5,23)	11 (9,13)	0.389
MII	12 (3, 20)	8 (7, 9)	0.196
2PN	11 (3,16)	3.5 (3,7)	0.181
Biopsied blastocytes	4 (3.5, 4.25)	2 (1.25,3.5)	0.155
Embryo suitable for transfer	1.5 (0,2.25)	0 (0,0.75)	0.405

### Patients follow-up and oncological outcome after IVF/PGT-M

Five Lynch syndrome patients had colonoscopy and transvaginal ultrasound (TVS) medium 12 months before PGT-M (range from 5-21 months) and 14.3 months after PGT-M/delivery(range from 10-18 months). Three Lynch syndrome patients got a new born after IVF/PGT-M. PGT-M1 had multiple colon polyps 6 months before ovarian stimulation, and find it recurrence 10 months after delivery by colonoscopy. PGT-M2-4, also received gynecologic ultrasound and colonoscopy before and after ovarian stimulation with no abnormalities detected. PGT-M4 suffered abnormal uterine bleeding after ovarian stimulation for 1 month, and received endometrium biopsy showing proliferative endometrium. PGT-M5 also showed no abnormalities before and after PGT-M.

In patients with BRCA1/2 mutation, PGT-M6、8 and 9 had BI-RADS3 breast nodules before ovarian stimulation. They reexamined breast ultrasound/MR 3 to 10 months after PGT/delivery, the nodules size did not enlarge. PGT-M7 with HBOC received breast CT 7 months after her ovarian induction, which showed no recurrence.

The Two fertility preservation patients, FP1 received her follow-up examination half year after last chemotherapy and showed no recurrence. The other FP2 patient is still in her chemotherapy cycles (see **Table 4**).

**Table 4:** Patients oncological follow-up after IVF/PGT-M.

		Evaluation procedures	Lesions before treatment (cm)	lesions after IVF/PGT-M (cm)	Lesions after pregnancy (cm)
Lynch syndrome	PGT-M1	Colonoscopy, TVS	Multiple colon polyps		Multiple colon polyps recurrence
	PGT-M2	Colonoscopy, TVS	None		None
	PGT-M3	Colonoscopy CT, TVS	None		None
	PGT-M4	Colonoscopy CT, TVS	None	None/abnormal uterine bleeding after IVF/PGT-M, endometrial biopsy showed proliferative endometrium	
	PGT-M5	Colonoscopy CT, TVS	None	None	
BRCA mutation	PGT-M6	Breast ultrasound	Breast nodules(BI-RADS3) 1.25*0.41cm	Breast nodules(BI-RADS3) 1.29*0.40cm	
	PGT-M7	Breast CT	None	None	
	PGT-M8	Breast ultrasound	Breast nodules(BI-RADS3) 0.3cm		Breast nodules(BI-RADS3) 0.3cm
	PGT-M9	Breast MR	0.5cm		0.5cm

TVS, transvaginal ultrasound.

### Ovarian stimulation complications

There were no complications like ovarian hyperstimulation syndrome after ovarian stimulation due to lower Gn dose and the use of letrozole.

## Discussion

Genomic sequencing is becoming more and more affordable, leading to an increasing demand for genetic testing as well as PGT-M. PGT-M provides a new opportunity to improve the fertility preservation and genetic blockade among patients with hereditary tumors caused by a single gene mutation. To date, knowledge of how PGT-M influences hormone-related diseases is limited. It is unclear whether IVF/PGT-M affects tumor progression. In this study, we retrospectively analyzed the records of PGT-M outcomes of hormone-related hereditary tumors in our reproductive center, including Lynch syndrome, and HBOC. A total of 32 embryos underwent PGT-M, and 9 (28.1%) embryos were suitable for transfer. Six transfer cycles were performed, and 5 cycles got clinical pregnancy (83%) with five newborns (83%). All the patients were not diagnosed with infertility, which may result in the higher pregnancy rate and live birth rate than reported (14).There was no significant difference of AMH and age between carriers and morbidities. The number of oocytes retrieved, MII, 2PN and biopsied blastocytes of morbidities seemed higher than those of carriers, but no obvious statistical difference was found due to the small sample size. The use of Letrozole(LE) in the process of ovulation induction may affect the number of oocytes and blastocysts obtained among morbidities.

We also analyzed the effect of IVF/PGT-M on tumor progression in patients with hormone-related diseases. In PGT-M cycles, more dosage of Gn would be used to harvest more oocytes and biopsied blastocytes than in IVF cycles. The total dose of Gn and the number of stimulation days in this study are similar to those reported in the literature (15). In our study, the HBOC patients who underwent PGT-M and fertility preservation showed no recurrence till now. In previous studies, Inge et al (11) showed that ovarian stimulation for BRCA1/2 mutation carriers did not increase breast cancer risk. The study included 2514 BRCA1/2 mutation carriers; 76(3%) patients received ovarian stimulation for IVF. A total of 938 (37.3%) BRCA1/2 mutation carriers developed breast cancer. Statistical analyzes showed ovarian stimulation did not increase the risk of breast cancer (HR: 0.79, 95% CI: 0.46–1.36). Kotsopoulos et al. (16) compared 1380 women with BRCA1/2 mutation with breast cancer history to 1380 women with BRCA1/2 mutation carriers without breast cancer history and found no difference between exposure to ovarian stimulation and breast cancer risk (OR: 0.98, 95% CI: 0.39–2.45), while exposing to gonadotropin-containing fertility treatment showed a nonsignificant increase risk of breast cancer (OR: 2.32, 95% CI: 0.91–5.95). Tamoxifen (TMXF)/LE alone or combined with low-dose Gn ovulation induction can reduce the amount of Gn in the cycle. Using LE after-oocyte retrieval reduced the risk of breast cancer compared with the natural cycle (17).

There was limited data about the effect of IVF on Lynch syndrome patients. Only one Lynch syndrome patient had recurrence of multiple colon polyps, the gynecologic ultrasound and coloscopy of the other four Lynch syndrome patients showed no abnormalities 10-18 months after IVF/pregnancy and further follow-up continued. For lynch syndrome patients, most guidelines recommend high definition screening colonoscopies in dedicated centers, starting at the age of 20–25 years old, with a surveillance interval of 1–2 years (18). Mandy Spaan et al. (19) took a research among 19,158 women who received ovarian stimulation for IVF (IVF group) with a median follow-up of 21 years. IVF group do not have an increased risk for colorectal cancer compared with the general population, but their risk is increased compared with women who received subfertility treatments other than IVF. Von Wolff et al. (20) reported that controlled ovarian hyperstimulation before tumor treatment does not affect the long-term survival time of patients. Ovulation-inducing drugs, including clomiphene citrate (CC), LE, TMXF, and Gn, do not increase the risk of gynecological malignancies. However, when CC dose is over 2000mg, and the duration is longer than 7 cycles, CC may increase the risk of ovarian cancer and endometrial carcinoma (21, 22). Long-term high-dose application of CC may increase the risk of endometrial cancer (23). Therefore, CC should be avoided in ovulation induction programs for endometrial cancer patients.

In our study, six embryo transfers were completed after PGT-M, resulting in five live births. One cycles used artificial cycles for endometrial preparation, and five used natural cycles. Patients of gynecological tumors should prioritize natural cycles for endometrial preparation, especially for breast cancer patients. If follicular growth is not satisfactory, endometrial preparation can be performed with TMXF alone or combined with low-dose human menopausal gonadotropin(HMG) to reduce estrogen levels to those of the natural cycle (24). For estrogen-independent diseases like cervical cancer, hormone replacement therapy (HRT) protocol does not increase the risk of recurrence (25). In patients with ovarian cancer, an HRT protocol is recommended considering impaired ovarian function after cancer treatment.

Studies on the time suitable for PGT-M after cancer therapy are limited. BRCA mutation carriers and Lynch syndrome patients may have a shortened reproductive window due to cancer diagnosis and treatment at young age and/or prophylactic adnexectomy. Two Lynch syndrome patients had colectomy surgery with or without chemotherapy 3 years before PGT-M. The HBOC patient came for PGT-M five years after breast surgery. The two HBOC patients for fertility preservation came to the doctor immediately after breast surgery before chemotherapy. In Lee’s study ^[23]^, 992 out of 31761 Korean women, who were treated for primary breast cancer under age 45, got pregnant. The median time between surgery and conception was 1059 days. According to guidelines of ASCO (American Society of Clinical Oncology), patients of breast carcinoma *in situ* are suggested to begin conception plan after surgery and radiotherapy. The recommended time before conception for breast invasive cancer patients with negative and positive lymph nodes is 2 years and 5 years (26). For patients of colorectal cancer, the recommended time before conception is 2 to 5 years after reaching a clinical cure by surgery and chemotherapy/radiotherapy (26, 27). Again, pregnancy and IVF/PGT-M plan for hereditary tumor syndrome patients need individually counsel and closely follow-up.

However, more research efforts including multicenter studies are needed to evaluate PGT-M effect on hormone-related hereditary tumor syndrome due to the limited sample size and follow-up time. The effect of PGT-M on other hereditary tumor syndrome related to estrogen levels, such as neurofibromatosis, could also be studied in future. Besides, further fertility comparisons between carriers and tumor patients could also be carried out.

## Conclusion

This study analyzed PGT-M data of hormone-related heritage tumor patients, to improve the consultation for their fertility and reproductive options. IVF/PGT-M treatment for women with hormone-related hereditary tumor syndrome is NOT associated with tumor progression in patients with Lynch syndrome and HBOC.

## Data availability statement

The original contributions presented in the study are included in the article. Further inquiries can be directed to the corresponding author.

## Ethics statement

The studies involving humans were approved by the Ethics Committee of Peking University Third Hospital with No. 2008013. The studies were conducted in accordance with the local legislation and institutional requirements. Written informed consent for participation was not required from the participants or the participants’ legal guardians/next of kin in accordance with the national legislation and institutional requirements.

## Author contributions

DW: Writing – original draft, Formal analysis, Data curation. XS: Writing – review & editing, Validation, Supervision, Methodology, Conceptualization. XZ: Writing – review & editing, Data curation. LY: Writing – review & editing, Supervision. XZ: Writing – review & editing, Validation. JY: Writing – review & editing, Validation. HL: Writing – review & editing, Validation. JQ: Writing – review & editing, Validation, Supervision.
